# Exposure to extreme heat and precipitation events associated with increased risk of hospitalization for asthma in Maryland, U.S.A.

**DOI:** 10.1186/s12940-016-0142-z

**Published:** 2016-04-27

**Authors:** Sutyajeet Soneja, Chengsheng Jiang, Jared Fisher, Crystal Romeo Upperman, Clifford Mitchell, Amir Sapkota

**Affiliations:** Maryland Institute for Applied Environmental Health, University of Maryland School of Public Health, 2234F SPH Building #255, College Park, 20742 MD USA; Department of Epidemiology, University of Maryland School of Public Health, College Park, 20742 MD USA; Maryland Department of Health and Mental Hygiene, Prevention and Health Promotion Administration, Baltimore, MD USA

**Keywords:** Asthma, Climate change, Extreme weather, Heat, Hospitalization, Precipitation, Respiratory illness

## Abstract

**Background:**

Several studies have investigated the association between asthma exacerbations and exposures to ambient temperature and precipitation. However, limited data exists regarding how extreme events, projected to grow in frequency, intensity, and duration in the future in response to our changing climate, will impact the risk of hospitalization for asthma. The objective of our study was to quantify the association between frequency of extreme heat and precipitation events and increased risk of hospitalization for asthma in Maryland between 2000 and 2012.

**Methods:**

We used a time-stratified case-crossover design to examine the association between exposure to extreme heat and precipitation events and risk of hospitalization for asthma (*ICD-9* code 493, *n* = 115,923).

**Results:**

Occurrence of extreme heat events in Maryland increased the risk of same day hospitalization for asthma (lag 0) by 3 % (Odds Ratio (OR): 1.03, 95 % Confidence Interval (CI): 1.00, 1.07), with a considerably higher risk observed for extreme heat events that occur during summer months (OR: 1.23, 95 % CI: 1.15, 1.33). Likewise, summertime extreme precipitation events increased the risk of hospitalization for asthma by 11 % in Maryland (OR: 1.11, 95 % CI: 1.06, 1.17). Across age groups, increase in risk for asthma hospitalization from exposure to extreme heat event during the summer months was most pronounced among youth and adults, while those related to extreme precipitation event was highest among ≤4 year olds.

**Conclusion:**

Exposure to extreme heat and extreme precipitation events, particularly during summertime, is associated with increased risk of hospitalization for asthma in Maryland. Our results suggest that projected increases in frequency of extreme heat and precipitation event will have significant impact on public health.

**Electronic supplementary material:**

The online version of this article (doi:10.1186/s12940-016-0142-z) contains supplementary material, which is available to authorized users.

## Background

The U.S. Centers for Disease Control and Prevention (CDC) reports that 25.5 million Americans are living with asthma, with approximately 439,000 cases resulting in hospitalization annually [1]. Annual costs to the U.S. economy, including loss of productivity, medical expenses, and premature death, is estimated to be $56 billion USD [2]. In the state of Maryland, over 430,000 adults were living with asthma (9.4 % prevalence) in 2013 [3], with a total annual hospitalization cost exceeding $66 million USD [4]. An extensive body of literature has shown that exposures to environmental risk factors such as indoor/outdoor allergens (i.e., mold, dander, dust mites, pollen), tobacco smoke, occupational hazards, and air pollution can exacerbate asthma [5–13].

More recent studies have investigated the association between meteorological parameters (e.g., temperature, precipitation) and risk of hospitalization for respiratory health outcomes, including asthma [14–20]. These studies have revealed that particular subpopulations, such as the elderly, are disproportionately impacted [17–19]. Changes in temperature can lead to airway obstruction that are associated with asthma attacks, alter onset and length of pollen season, and increase pollen production [21–25]. In addition to temperature, precipitation may also influence the risk of asthma attacks. In particular, several studies have highlighted elevated risk of asthma attacks following major thunderstorms [26–29]. Thunderstorm events, or periods of heavy rainfall and intense wind, are believed to trigger the release of fungal spores that are carried by wind, thereby resulting in increased exposure to these triggers [26–28].

An increasing body of literature, including the most recent Intergovernmental Panel on Climate Change (IPCC) report, suggest that extreme weather events are projected to increase in frequency, intensity, as well as duration in the coming decades in response to our changing climate [30]. While previous studies have investigated the link between daily weather (temperature, precipitation, and thunderstorm) and asthma morbidity, there is limited data regarding how increased frequency of extreme events may impact people that are living with asthma. In this study, we identified extreme heat and precipitation events using location and calendar day specific climatology data based on a 30-year baseline (1960–1989), and examined the associations between the frequency of such extreme events and risk of hospitalization for asthma in the state of Maryland during the 2000 to 2012 period. We further investigated how this association differs across various demographic subgroups (i.e., age groups, gender, and race/ethnicity) in Maryland with a particular emphasis on the summertime exposure.

## Methods

### Extreme heat and precipitation events

Extreme heat and precipitation events during the study period (2000–2012) were identified as previously described, with slight modifications [31, 32]. In brief, meteorological data (Additional file 1: Figure SA.1) was obtained from the National Climatic Data Center [33] for the period of 1960–2012. Using daily maximum temperature (TMAX) and total precipitation (PRCP) for the 1960 to 1989 period, county-specific 30-year baselines for TMAX and PRCP for a given calendar day were determined using a 31-day window that centered on the particular calendar day. For example, to compute Garrett County’s July 15^th^ baseline TMAX and PRCP, we compiled all daily TMAX and PRCP values for Garrett County from July 1^st^ to July 31^st^ between 1960 and 1989. We then identified the 95^th^ percentile (TMAX) and 90^th^ percentile (PRCP) value of this distribution and referred to them as Extreme Temperature Threshold 95^th^ percentile (ETT95), and Extreme Precipitation Threshold 90^th^ percentile (EPT95), respectively. Daily TMAX and PRCP values for each counties during the study period (2000–2012) were compared to their respective daily ETT_95_ and EPT_90_ and assigned a value of “1” if they exceeded the threshold and identified as an extreme event (of heat or precipitation). This data was linked to the asthma hospitalization data based on the county of residence.

### Hospitalization data

We obtained inpatient hospital admission data for asthma (*International Classification of Diseases, Ninth Revision* (*ICD-9*) principal diagnosis code: 493) from the Maryland Department of Health and Mental Hygiene. Hospitalization admissions spanned January 1, 2000 to December 14, 2012 for the entire state of Maryland. For each record, additional information was extracted including county of residence, age, gender, race/ethnicity, and hospital admission date. The institutional review board at the University of Maryland, College Park as well as the Maryland Department of Mental Health and Hygiene approved acquisition and use of all data.

### Statistical analysis

A time-stratified case-crossover analysis [34] was utilized to assess the association between exposure to extreme events and the risk of hospitalization for asthma. For selection of control periods, the study time frame (2000–2012) was divided into consecutive 28-day intervals. For each hospitalization admission date, three control days matched by day of week occurring on 7, 14, or 21 days before, after, or a combination of both around the admission day were assigned within the same interval. Conditional logistic regression models, performed in SAS (Version 9.3, Cary, NC), were used to calculate association between exposure to extreme heat and precipitation event and risk of same day (lag 0) hospitalization for asthma. In an overall analysis, we investigated the association between exposure to extreme heat and precipitation events and risk of hospitalization for asthma. We further stratified the analysis to investigate how the risk associated with extreme heat and precipitation events varied across season (spring, summer, autumn, winter), age (≤4, 5 to 17, 18 to 64, ≥65), race/ethnicity (non-Hispanic white, non-Hispanic black, and Hispanic), and gender groups since prior studies have shown the risk to differ across these factors [13, 14, 17].

Additional sensitivity analyses were conducted examining lag periods (1 day, 2 day, and 0–2 days) and combinations of the thresholds utilized for extreme heat and precipitation (90^th^, 95^th^, and 99^th^ percentiles).

## Results

There were a total of 115,923 inpatient cases of hospitalization for asthma in Maryland from 2000 to 2012 (Table 1). Autumn had the highest percentage of cases (29 %) and summer the least (18 %). The median age with interquartile range (IQR) for all cases was 44 years of age (12–60) (Table 1). Considerable seasonal variability was observed across all age groups. For example, compared to summer, autumn had 131 and 267 % higher hospital admissions for the ≤4 and 5 to 18 years subgroups. However, increases were considerably lower (32 and 14 %) for the 18 to 64 and ≥65 years of age groups.

**Table 1:** Demographic Characteristics of Hospitalizations for Asthma in Maryland: 2000 to 2012

	Hospitalizations-No. (%)				
	Segmented by Season^a^				
Characteristic	Winter (*n* = 30,436)	Spring (*n* = 31,103)	Summer (*n* = 20,776)	Autumn (*n* = 33,608)	Total (*n* = 115,923)
Gender^b^					
Female	19,326 (64)	18,954 (61)	13,081 (63)	19,334 (58)	70,695 (61)
Male	11,110 (36)	12,148 (39)	7,695 (37)	14,273 (42)	45,226 (39)
Age at hospitalization, median (IQR), yrs^c^	47 (20–62)	44 (12–60)	47 (27–61)	37 (7–55)	44 (12–60)
Age Group^d^					
≤4	4,397 (15)	4,841 (16)	2,663 (13)	6,142 (18)	18,043 (16)
5 to 17	3,350 (11)	4,669 (15)	1,846 (9)	6,784 (20)	16,649 (14)
18 to 64	16,256 (53)	15,400 (50)	11,993 (58)	15,813 (47)	59,462 (51)
≥65	6,433 (21)	6,192 (20)	4,274 (21)	4,869 (15)	21,768 (19)
Race/Ethnicity					
Non-Hispanic White	13,320 (44)	12,930 (42)	8,132 (39)	12,769 (38)	47,151 (41)
Non-Hispanic Black	14,513 (48)	15,296 (49)	10,913 (53)	17,625 (52)	58,347 (50)
Hispanic	754 (3)	879 (3)	446 (2)	968 (3)	3,047 (3)
Other	860 (3)	957 (3)	545 (3)	1,117 (3)	3,479 (3)
Unknown	989 (3)	1,041 (3)	740 (4)	1,129 (3)	3,899 (3)

*Abbreviations*: *IQR* Interquartile Range, *yrs* years

^a^Seasons are winter (December-February), spring (March-May), summer (June-August), and autumn (September-November)

^b^Unknown (*n* = 1) for spring season; unknown (*n* = 1) for autumn season

^c^2,604 age observations were excluded due to age <1 year old, and were thus unknown

^d^Unknown (*n* = 1) for spring season

**Fig. 1:**
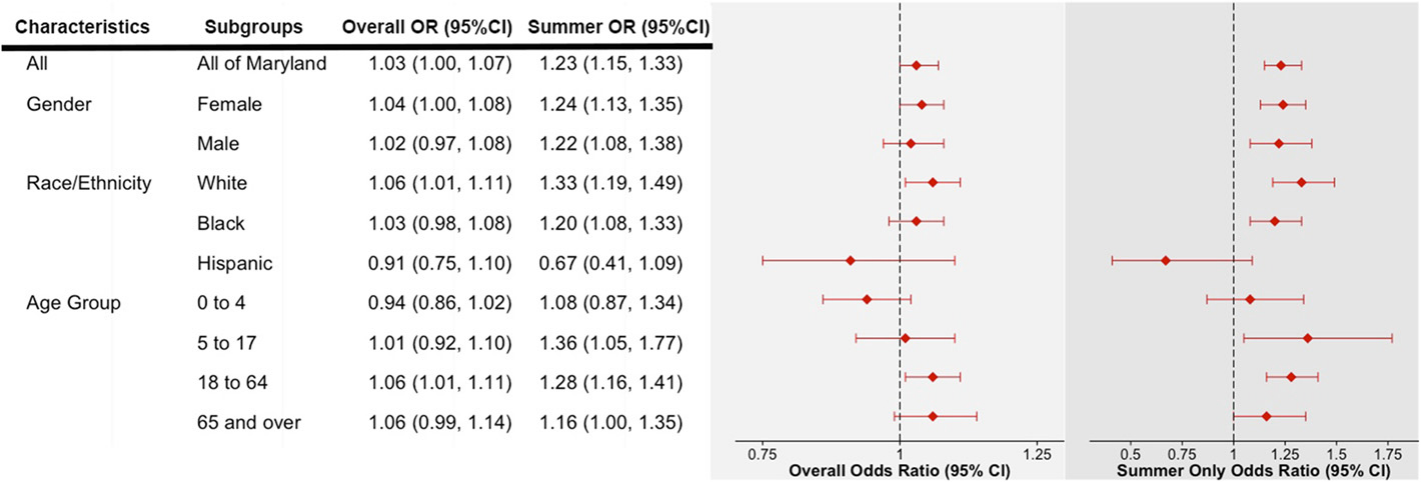
Odds Ratios (ORs) and 95 % Confidence Interval (95 % CI) for Exposure to Extreme Heat Events and Risk of Hospitalization for Asthma in Maryland Between 2000 and 2012, adjusted for extreme precipitation event

The association between exposure to extreme heat event and increased risk of hospitalization for asthma is shown in Fig. 1 for the 2000 to 2012 period overall, and for summer months during the same period. In the overall analysis, exposure to extreme heat event in Maryland was associated with a 3 % increase in the risk of hospital admission for asthma (Odds Ratio (OR): 1.03, 95 % Confidence Interval (95 % CI): 1.00, 1.07). This risk was considerably higher when the analysis was restricted to the summer season (OR: 1.23, 95 % CI: 1.15, 1.33), with a noted heterogeneity between seasons (results not shown). This trend of higher risk during summer months was consistent across gender, age groups, and race, with a noted exception observed for Hispanics (Fig. 1). Among race groups, the risk of hospitalization for asthma associated with summertime extreme heat event was highest for non-Hispanic whites (OR = 1.33, 95 % CI: 1.19, 1.49), followed by non-Hispanic blacks (OR: 1.20, 95 % CI: 1.08, 1.33). Such increases in risk were not observed among Hispanics (OR: 0.67, 95 % CI: 0.41, 1.09). Likewise, the increase in risk associated with summertime extreme heat event was highest among the 5 to 17 years age group (OR: 1.36, 95 % CI: 1.05, 1.77).

**Fig. 2:**
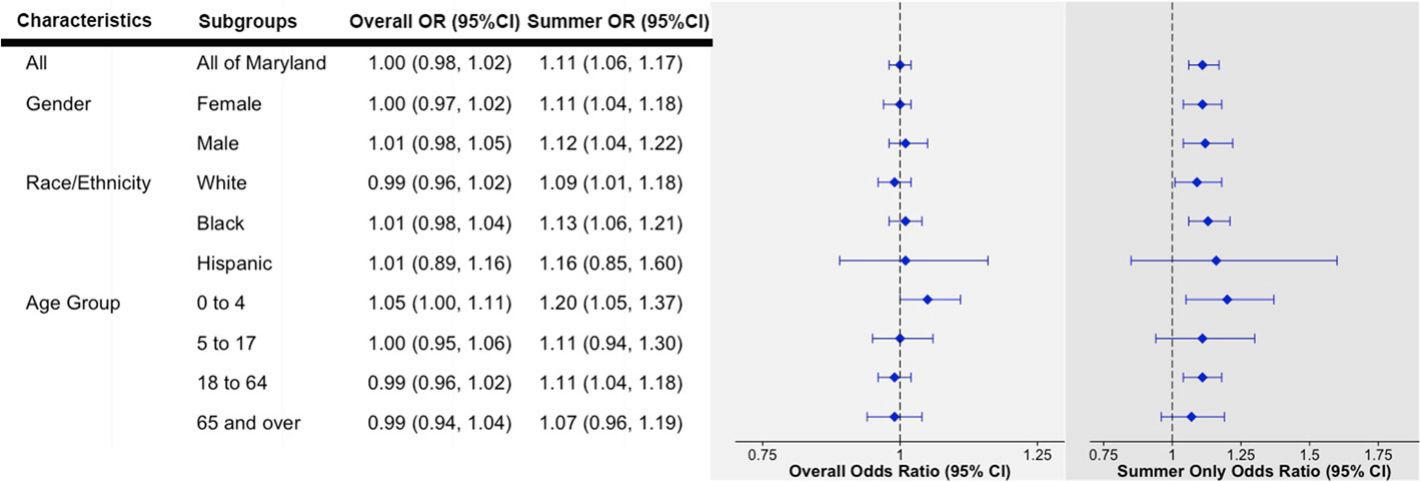
Odds Ratios (ORs) and 95 % Confidence Interval (95 % CI) for Exposure to Extreme Precipitation Events and Risk of Hospitalization for Asthma in Maryland Between 2000 and 2012, adjusted for extreme heat events

The corresponding results for exposure to extreme precipitation events, which were included in the same model as extreme heat, and risk of hospitalization for asthma is depicted in Fig. 2. In the overall analysis, extreme precipitation was not associated with an increased risk of hospitalization for asthma (OR: 1.00, 95 % CI: 0.98, 1.02). However, during the summer months, exposure to extreme precipitation event was associated with an 11 % increase in risk of hospitalization for asthma (OR: 1.11, 95 % CI: 1.06, 1.17). The risk associated with the summertime extreme precipitation event was similar for gender (male vs. female) and race/ethnicity (non-Hispanic blacks, non-Hispanic whites, and Hispanics), although the increased risk was not statistically significant among Hispanics. Across age groups, we observed slightly higher risk among the youngest age group (≤4 years of age OR: 1.20, 95 % CI: 1.05, 1.37) compared to the other 3 subgroups (5 to 17 years of age OR: 1.11, 95 % CI: 0.94, 1.30; 18 to 64 years of age OR: 1.11, 95 % CI: 1.04, 1.18; and ≥ 65 years of age OR: 1.07, 95 % CI: 0.96, 1.19).

The results of the sensitivity analyses, presented in the appendix), using different lag structures (Additional file 1: Table SA.1) did not change the results substantially. We have provided results from additional sensitivity analysis (Additional file 1: Table SA.2), where extreme events were identified using different thresholds (eg 90^th^, 95^th^, and 99^th^ percentiles). This did not change our overall conclusions. Also, findings for other seasons are presented within the appendix (Additional file 1: Table SA.3).

## Discussion

Previous studies have shown that the frequency, intensity, as well as the length of extreme events will continue to increase in the foreseeable future in response to a changing climate [30]. In this study, we investigated how increases in frequency of extreme heat and precipitation events are related to the risk of hospitalization for asthma in Maryland. Such information is needed for estimating projected rise in disease burden in the future in response to the increases in frequency of extreme events, as well as to inform local adaptation strategies. Our results show that exposure to extreme heat events is associated with an elevated risk of hospitalization for asthma in Maryland. Increases in risk were considerably higher for summertime extreme heat events. Exposure to extreme precipitation events was not associated with an elevated risk of hospitalization for asthma in our overall analysis with a noted exception for those that were very young (≤4 years of age). However, we did observe consistently higher and statistically significant risks of hospitalization for asthma associated with summertime extreme precipitation events in most subgroups.

Summer months are associated with higher concentration of many important pollutants, such as ground level ozone, that are related to airway inflammation, asthma exacerbation [35–37], and other acute respiratory outcomes [38–41]. Previous studies have shown that higher temperature contributes to elevated ozone concentrations [9, 38]. Therefore, the observed increased risk of hospitalization for asthma associated with the exposure to extreme heat events may be explained, in part, by exposure to ground-level ozone. This is supported by the fact that the increases in summertime risk are more pronounced in children 5 to 17 years of age followed by adults 18 to 64 years of age, two groups that we expect would be the most active, but not in the very young of 0 to 4 years of age. The absence of risk observed among Hispanics may be due to very small sample size for this particular subgroup (*n* = 446), so findings for this subgroup should be interpreted cautiously.

In addition to temperature, precipitation events have been shown to influence asthma attacks [26–28, 42]. Heavy rainfall may facilitate the release of allergens (e.g., spore plumes and pollen), that contribute to asthma exacerbations [43–47]. Like rainfall, thunderstorms may also contribute to the release of pollen, particularly grass pollen, and thereby contribute to the “thunderstorm asthma” phenomenon [27, 28, 42, 43]. Furthermore, heavy rainfall events could alter the humidity in the environment and contribute to changes in airflow in the respiratory system that could further exacerbate asthma [23, 48, 49]. While our study did not specifically classify extreme precipitation events as a thunderstorm event, it is conceivable that there is significant overlap between the two. The fact that extreme precipitation related risk was evident only during summertime is supported by the previous studies that have tied thunderstorm asthma with grass pollen [27, 28, 42, 43]. Our findings regarding the most consistent increase in risk of hospitalization for asthma related to extreme precipitation events among the youngest age group is in agreement with previous literature that have reported similar findings for thunderstorm-associated asthma [43]. Mounting evidence suggests that this age group may be susceptible to allergen sensitization and allergic disease induction [50].

This study utilized exposure metrics that were derived based on three decades (1960–1989) of baseline data and was specific to each calendar day and county. Furthermore, the hospitalization data encompassed a reasonably long time frame (13 years), which included considerable variability in both the exposures and outcome data. The analysis controlled for confounding at the individual level by utilizing the case-crossover design [34, 51]. There are some limitations of the study that merit further discussion. The exposure metric used was dichotomous in nature, and did not account for the intensity as well as duration of the event. Also, as the spatial resolution of our exposure metric was county level, there is a potential for exposure misclassification as it does not distinguish heterogeneity in exposure within county, particularly larger counties that have both rural and urban areas. Future research needs to consider finer spatial resolution, such as ZIP code or census track. Our geographic area was relatively small as well (24 counties in Maryland) and the ability to distinguish between recurrent hospitalizations and information on duration of stay were unavailable for this study. Use of the ICD code for asthma diagnosis could be a point of concern for those less than 4 years of age as diagnosing asthma in that age category can be difficult [52], thus future research should seek to conduct further analysis for this age group using different classification methods.

As more and more people are living longer, often with chronic diseases such as asthma, understanding how such vulnerable populations will be differentially impacted by increases in the frequency of extreme events that are projected to rise in response to our changing climate is a public health priority. Building effective adaptation strategies that are based on disease forecast models and early warning systems require an in-depth understanding of the exposure response relationships as well extreme event projections that may not be uniform across geographic regions. Our findings build upon the previous literature related to meteorological parameters (maximum daily temperature, precipitation) and asthma exacerbation by providing exposure response functions for exposure to extreme events and increased risk of hospitalization for asthma. These findings are relevant in the context of our changing climate where such events are expected to rise in frequency, duration, and intensity.

## Conclusion

Our findings suggest that exposures to increased frequency of extreme heat and precipitation events are associated with elevated risk of hospitalization for asthma in Maryland, particularly during summertime. While summertime extreme heat event related risk is most pronounced among youths and adults, extreme precipitation event related risk is most pronounced among the youngest age group (≤4 years of age). Local adaptation strategies need to account for such differential vulnerability.

## Additional file

Additional file 1: Supplemental Materials. **Table A.1.** Sensitivity Analysis Showing the Impact of Lag Structures for the Odds Ratios and 95 % Confidence Intervals. **Table A.2.** Sensitivity analysis across extreme threshold combinations for overall model for the entire state of Maryland. **Table A.3.** Analysis Showing the Odds Ratios and 95 % Confidence Intervals for Exposure to Extreme Events for the Spring, Winter, and Autumn seasons. **Figure A.1.** Location of weather stations in Maryland. **Figure A.2.** Monthly averaged number of extreme heat and extreme precipitation events by county, for overall years and during summer months only (2000–2012). (DOCX 62764 kb)

## Abbreviations

95 % CI: 95 % Confidence Interval
CDC: United States Center for Disease Control and Prevention
EPT_90_: Extreme Precipitation Threshold 90^th^ percentile
ETT_95_: Extreme Heat Threshold 95^th^ percentile
*ICD-9*: *International Classification of Diseases, Ninth Revision*
IPCC: United Nations Intergovernmental Panel on Climate Change
IQR: interquartile range
OR: Odds Ratio
PRCP: daily total precipitation
TMAX: maximum daily temperature (TMAX)
USD: United states Dollar
yrs: years
