# Supplementary material for: Exposure to extreme heat and precipitation events associated with increased risk of hospitalization for asthma in Maryland, U.S.A
Source: Environ Health. 2016 Apr 27;15:57. doi: 10.1186/s12940-016-0142-z (PMC4847234; doi:10.1186/s12940-016-0142-z)
Supplement: Additional file 1: — Supplemental Materials. Table A.1. Sensitivity Analysis Showing the Impact of Lag Structures for the Odds Ratios and 95 % Confidence Intervals. Table A.2. Sensitivity analysis across extreme threshold combinations for overall model for the entire state of Maryland. Table A.3. Analysis Showing the Odds Ratios and 95 % Confidence Intervals for Exposure to Extreme Events for the Spring, Winter, and Autumn seasons. Figure A.1. Location of weather stations in Maryland. Figure A.2. Monthly averaged number of extreme heat and extreme precipitation events by county, for overall years and during summer months only (2000–2012). (DOCX 62764 kb) [file 12940_2016_142_MOESM1_ESM.docx]

**Supplemental Materials**

| **Table A.1.** Sensitivity Analysis Showing the Impact of Lag Structures for the Odds Ratios and 95% Confidence Intervals. | | | | | |
| --- | --- | --- | --- | --- | --- |
| **Exposure metric** | **Model** | **Lag Structure (days)** | **Odds Ratio** | **95% Confidence Interval** |  |
|  |  |  |  |  |  |
| *Extreme Heat* | Overall | 1 | 1.04 | (1.01, 1.08) |  |
|  | Overall | 2 | 1.04 | (1.00, 1.07) |  |
|  | Overall | 0-2 | 1.03 | (1.01, 1.06) |  |
|  | Summer | 1 | 1.23 | (1.15, 1.32) |  |
|  | Summer | 2 | 1.17 | (1.09, 1.25) |  |
|  | Summer | 0-2 | 1.21 | (1.15, 1.28) |  |
| *Extreme Precipitation* | Overall | 1 | 0.99 | (0.97, 1.01) |  |
|  | Overall | 2 | 0.97 | (0.95, 1.00) |  |
|  | Overall | 0-2 | 1.00 | (0.98, 1.01) |  |
|  | Summer | 1 | 1.10 | (1.05, 1.15) |  |
|  | Summer | 2 | 0.96 | (0.91, 1.01) |  |
|  | Summer | 0-2 | 1.06 | (1.03, 1.10) |  |

Abbreviations: Lag 1 day: 1 day lag of extreme event; Lag 2 day: 2 day lag of extreme event; Lag 0-2 day: Presence of extreme event between day 0 and day 2.

**Table A.2.** Sensitivity analysis across extreme threshold combinations for overall model for the entire state of Maryland.

|  | **Extreme Event Threshold** | **Odds Ratio** | **95% LowerCL** | **95% UpperCL** | **Group** |
| --- | --- | --- | --- | --- | --- |
|  |  |  |  |  |  |
| Model 1 | Precipitation - 90th% | 1.00 | 0.98 | 1.03 | All of Maryland |
|  | Temperature - 90th% | 1.04 | 1.02 | 1.07 | All of Maryland |
|  |  |  |  |  |  |
| Model 2 | Precipitation - 90th% | 1.00 | 0.98 | 1.02 | All of Maryland |
|  | Temperature - 95th% | 1.03 | 1.00 | 1.07 | All of Maryland |
|  |  |  |  |  |  |
| Model 3 | Precipitation - 90th% | 1.00 | 0.98 | 1.02 | All of Maryland |
|  | Temperature - 99th% | 1.13 | 1.06 | 1.21 | All of Maryland |
|  |  |  |  |  |  |
| Model 4 | Precipitation - 95th% | 0.99 | 0.96 | 1.02 | All of Maryland |
|  | Temperature - 90th% | 1.04 | 1.02 | 1.07 | All of Maryland |
|  |  |  |  |  |  |
| Model 5 | Precipitation - 95th% | 0.99 | 0.96 | 1.02 | All of Maryland |
|  | Temperature - 95th% | 1.03 | 1.00 | 1.07 | All of Maryland |
|  |  |  |  |  |  |
| Model 6 | Precipitation - 95th% | 0.99 | 0.96 | 1.01 | All of Maryland |
|  | Temperature - 99th% | 1.13 | 1.06 | 1.21 | All of Maryland |
|  |  |  |  |  |  |
| Model 7 | Precipitation - 99th% | 0.96 | 0.91 | 1.02 | All of Maryland |
|  | Temperature - 90th% | 1.04 | 1.02 | 1.07 | All of Maryland |
|  |  |  |  |  |  |
| Model 8 | Precipitation - 99th% | 0.96 | 0.91 | 1.02 | All of Maryland |
|  | Temperature - 95th% | 1.03 | 1.00 | 1.07 | All of Maryland |
|  |  |  |  |  |  |
| Model 9 | Precipitation - 99th% | 0.96 | 0.91 | 1.02 | All of Maryland |
|  | Temperature - 99th% | 1.13 | 1.06 | 1.21 | All of Maryland |

**Table A.3.** Analysis Showing the Odds Ratios and 95% Confidence Intervals for Exposure to Extreme Events for the Spring, Winter, and Autumn seasons.

| **Time Period** | **Extreme Event Threshold** | **Odds Ratio** | **95% LowerCL** | **95% UpperCL** |
| --- | --- | --- | --- | --- |
|  |  |  |  |  |
| Spring | Precipitation - 90th% | 1.05 | 1.00 | 1.09 |
|  | Temperature - 95th% | 1.00 | 0.94 | 1.06 |
|  |  |  |  |  |
| Autumn | Precipitation - 90th% | 0.97 | 0.93 | 1.01 |
|  | Temperature - 95th% | 0.88 | 0.81 | 0.95 |
|  |  |  |  |  |
| Winter | Precipitation - 90th% | 0.92 | 0.88 | 0.96 |
|  | Temperature - 95th% | 1.05 | 0.99 | 1.10 |

**Figure A.1.** Location of weather stations in Maryland.


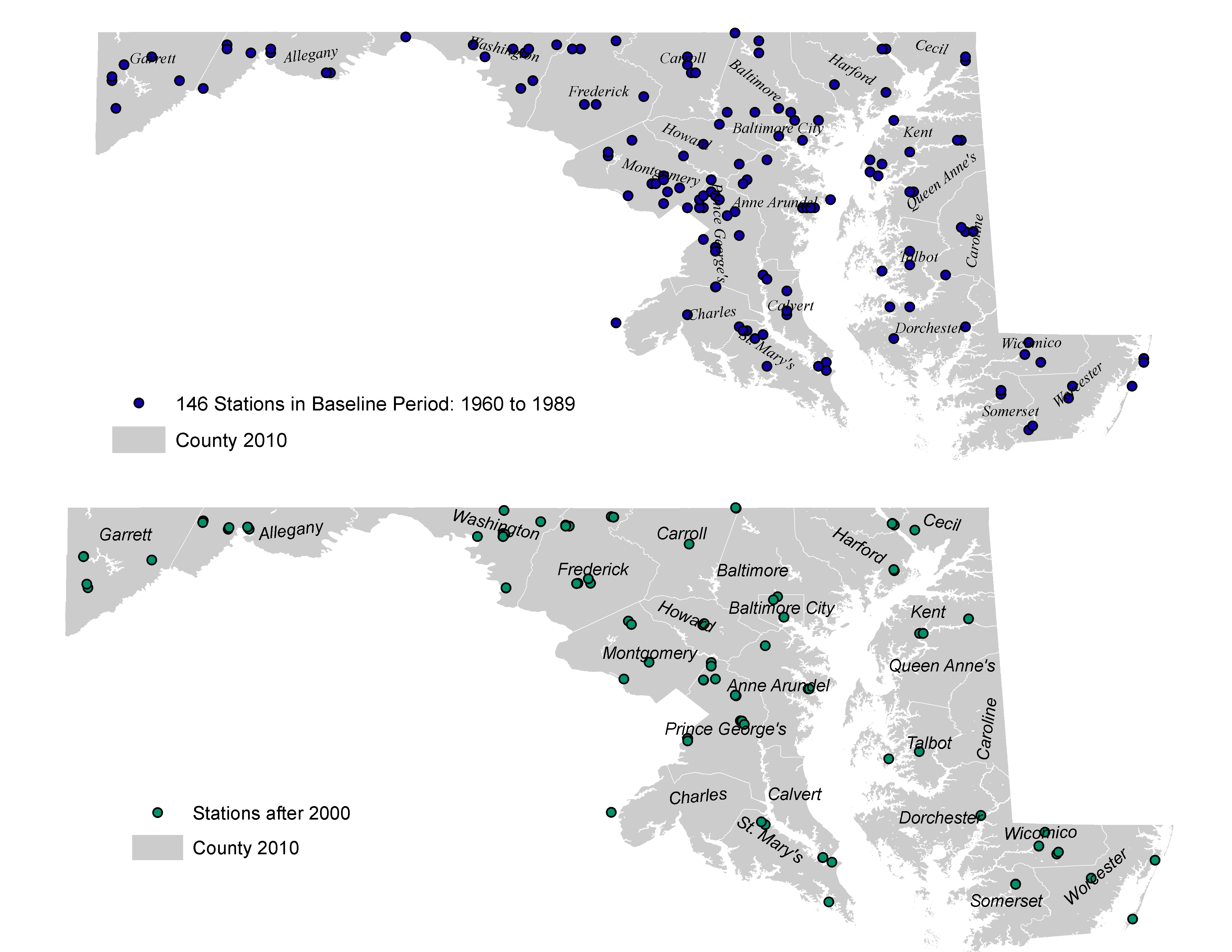


**Figure A.2.** Monthly averaged number of extreme heat and extreme precipitation events by county, for overall years and during summer months only (2000-2012).


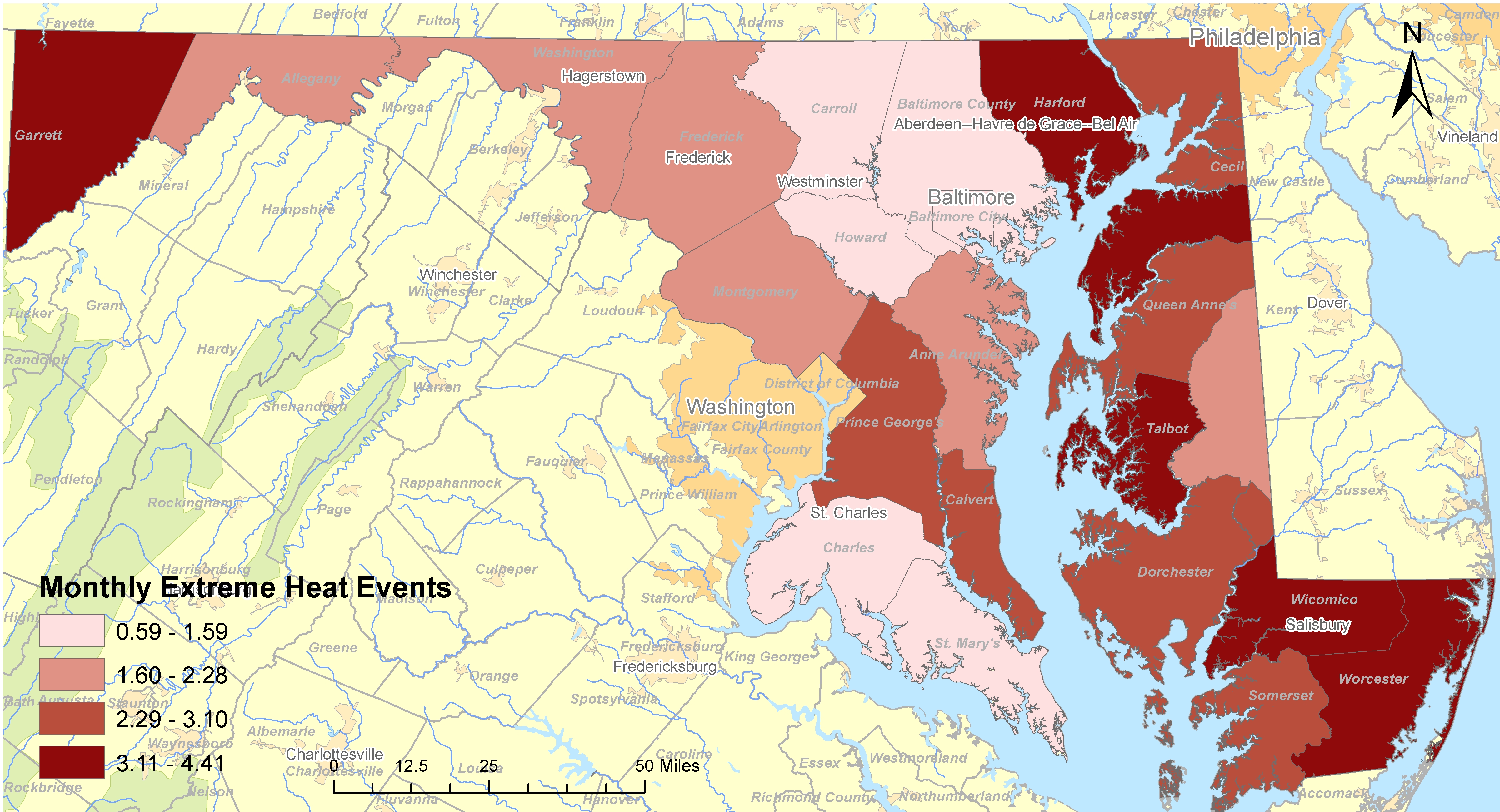

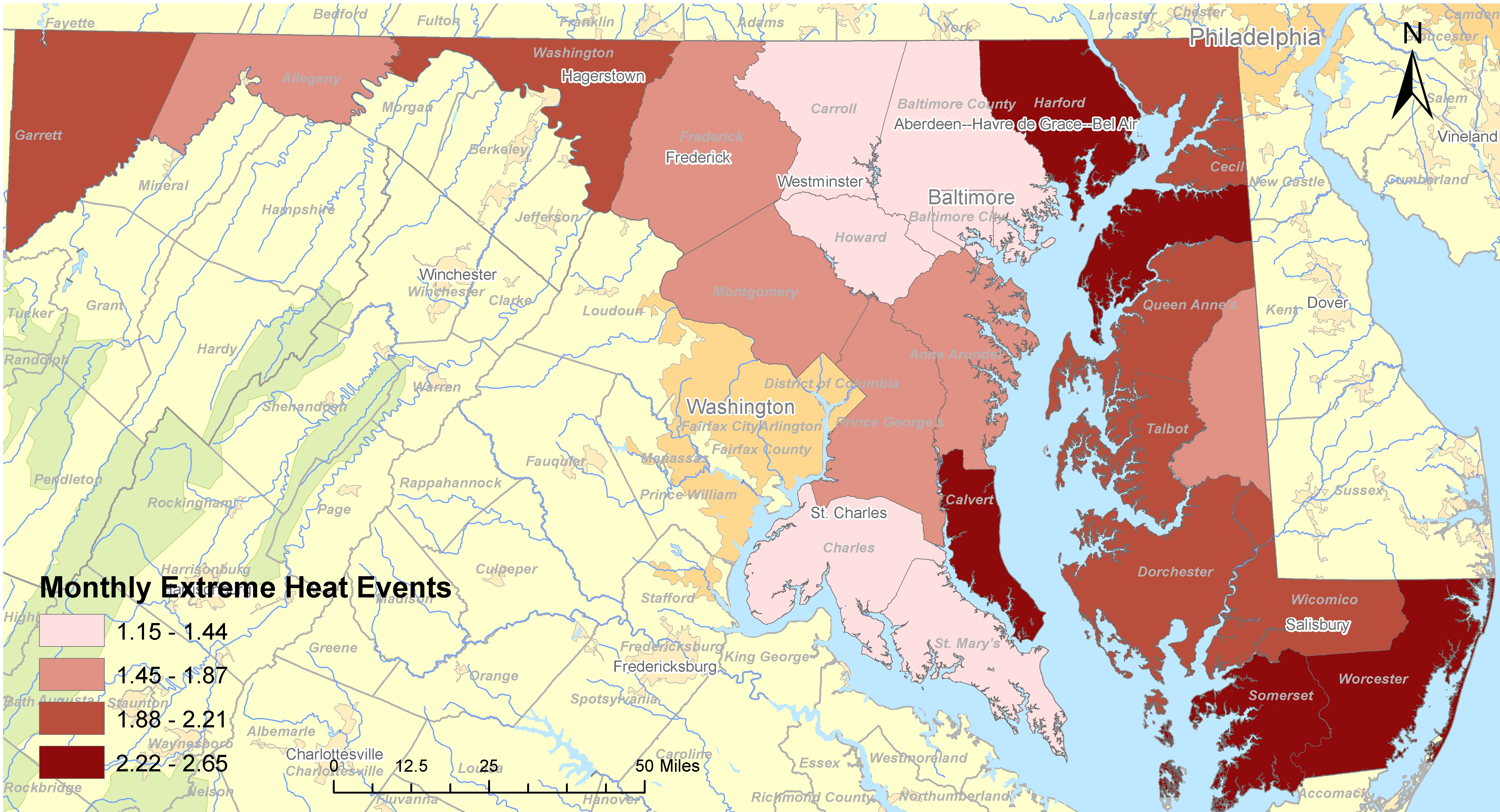

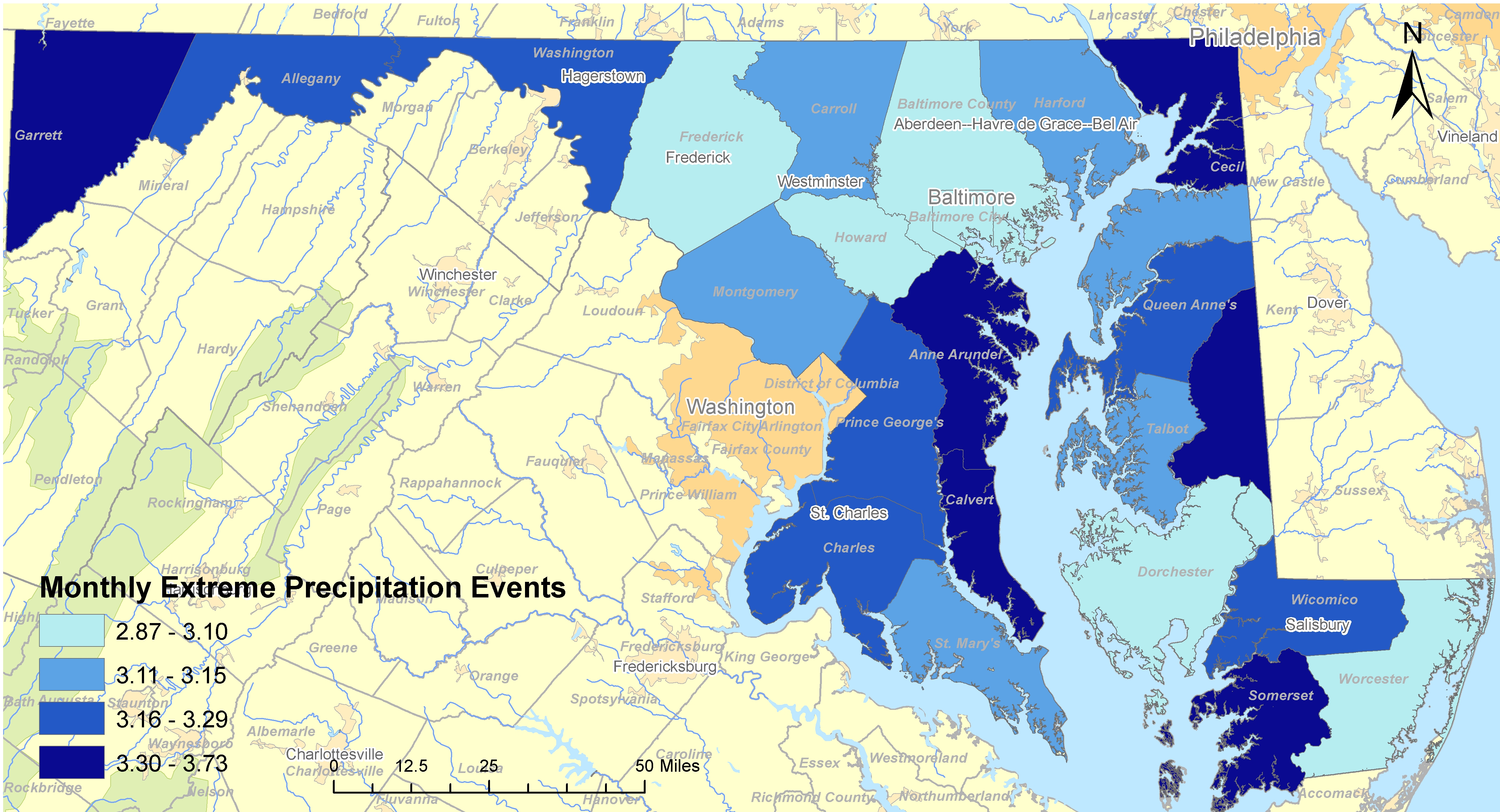

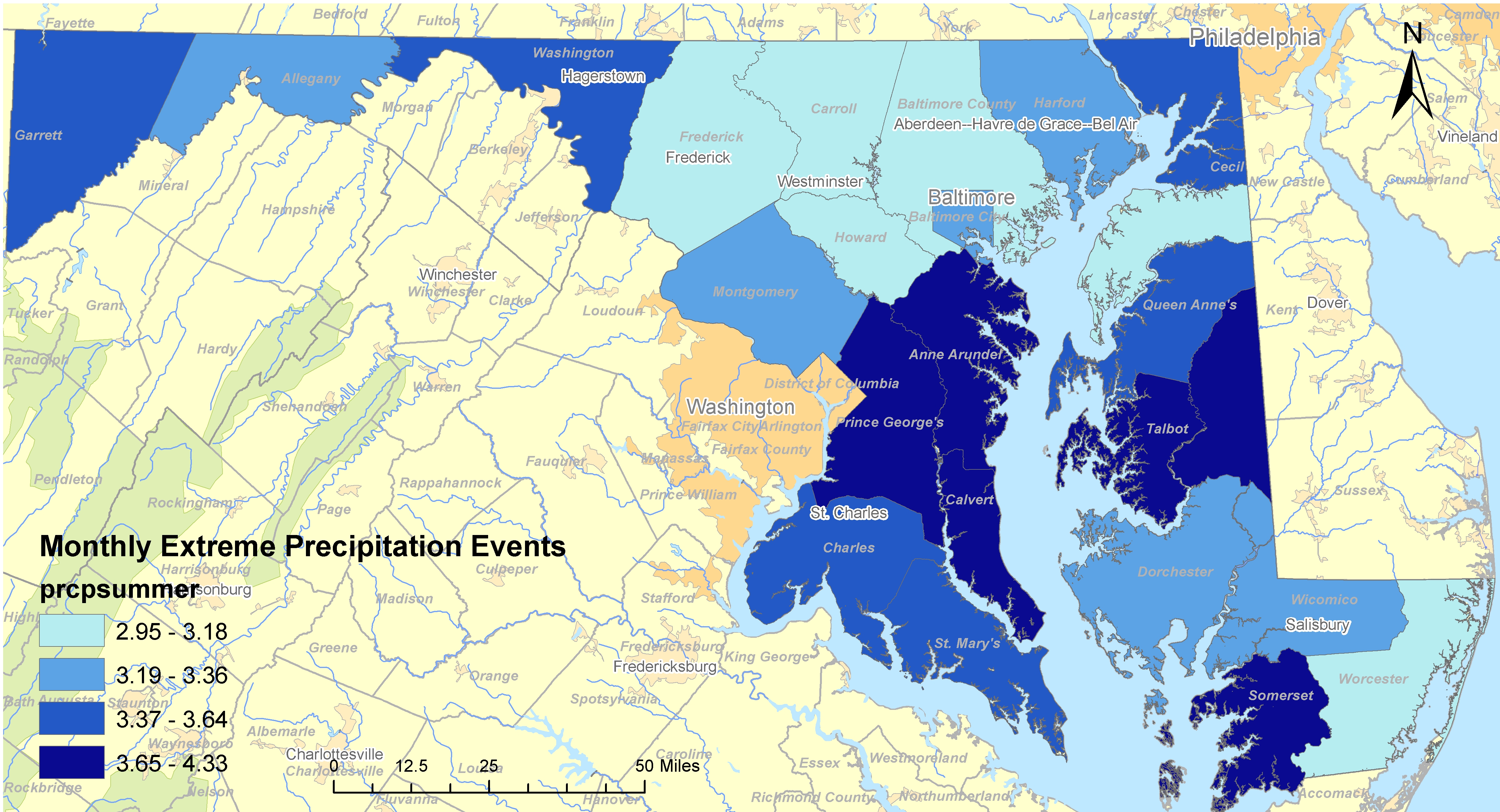


**Overall (2000-2012)**

**Summer months (2000-2012)**

**Extreme Heat**

**Extreme Precipitation**
